# Comparing the MRI-based Goutallier Classification to an experimental quantitative MR spectroscopic fat measurement of the supraspinatus muscle

**DOI:** 10.1186/s12891-016-1216-3

**Published:** 2016-08-22

**Authors:** Fabian Gilbert, Dirk Böhm, Lars Eden, Jonas Schmalzl, Rainer H. Meffert, Herbert Köstler, Andreas M. Weng, Dirk Ziegler

**Affiliations:** 1 Department of Trauma, Hand, Plastic and Reconstructive Surgery, Julius-Maximilians-University of Wuerzburg, Oberduerrbacherstr. 6, D-97080 Wuerzburg, Germany; 2 Ortho Mainfranken Wuerzburg, Bismarckstraße 16, D-97080 Wuerzburg, Germany; 3 Department of Radiology, Julius-Maximilians-University of Wuerzburg, Oberduerrbacherstr. 6, D-97080 Wuerzburg, Germany

**Keywords:** Rotator cuff, MRI, Spectroscopy, Goutallier Classification, Shoulder surgery

## Abstract

**Background:**

The Goutallier Classification is a semi quantitative classification system to determine the amount of fatty degeneration in rotator cuff muscles. Although initially proposed for axial computer tomography scans it is currently applied to magnet-resonance-imaging-scans. The role for its clinical use is controversial, as the reliability of the classification has been shown to be inconsistent. The purpose of this study was to compare the semi quantitative MRI-based Goutallier Classification applied by 5 different raters to experimental MR spectroscopic quantitative fat measurement in order to determine the correlation between this classification system and the true extent of fatty degeneration shown by spectroscopy.

**Methods:**

MRI-scans of 42 patients with rotator cuff tears were examined by 5 shoulder surgeons and were graduated according to the MRI-based Goutallier Classification proposed by Fuchs et al. Additionally the fat/water ratio was measured with MR spectroscopy using the experimental SPLASH technique. The semi quantitative grading according to the Goutallier Classification was statistically correlated with the quantitative measured fat/water ratio using Spearman’s rank correlation.

**Results:**

Statistical analysis of the data revealed only fair correlation of the Goutallier Classification system and the quantitative fat/water ratio with *R* = 0.35 (*p* < 0.05). By dichotomizing the scale the correlation was 0.72. The interobserver and intraobserver reliabilities were substantial with *R* = 0.62 and *R* = 0.74 (*p* < 0.01).

**Conclusion:**

The correlation between the semi quantitative MRI based Goutallier Classification system and MR spectroscopic fat measurement is weak. As an adequate estimation of fatty degeneration based on standard MRI may not be possible, quantitative methods need to be considered in order to increase diagnostic safety and thus provide patients with ideal care in regard to the amount of fatty degeneration. Spectroscopic MR measurement may increase the accuracy of the Goutallier classification and thus improve the prediction of clinical results after rotator cuff repair. However, these techniques are currently only available in an experimental setting.

## Background

Fatty degeneration (FD) of the rotator cuff muscles is observed after tendon rupture or nerve damage of the rotator cuff and has a major influence on the anatomical and clinical result after surgical repair [1, 2]. Tendon rupture leads to changes in the muscles physiology, structure and function, as the tensile forces decrease atrophy and fatty of the muscle occurs. This process has been termed fatty muscular degeneration [3, 4]. Severe preoperative FD results in high failure rates of rotator cuff repair and thus correlates with a poor functional outcome [5–7]. FD is an irreversible process even after successful rotator cuff repair a regeneration of the muscle tissue has not been observed [5, 6]. Therefore surgery should be performed before severe FD occurs [8–10]. However, FD has been shown to be partially reversible in a sheep model [11]. The amount of FD should be estimated preoperatively in a standardized classification as it is a key factor for the timing and the expectable clinical result after rotator cuff repair [5]. Computer tomography [CT] based grading of the FD was first suggested by Goutallier et al. in axial scans and was modified by Fuchs et al. in 1999 for magnetic resonance imaging [MRI] [12, 13]. The modified Goutallier Classification is a semi quantitative assessment with five grades and it has become the standard reference for estimating FD using oblique-sagittal t1-weighted MR-images [14].

The high impact of the FD on the postoperative result has led to numerous efforts to make the Goutallier Classification more reliable and valid. The interobserver reliability has been reported in multiple studies [15–18]. The reliability of the existing classification system is controversial and a further simplification of the classification of FD was suggested in order to increase reliability, nevertheless interobserver reliability continues to remain unsatisfactory [12, 14, 15, 17]. Thus objective methods, e.g. MR spectroscopy, may provide additional information and increase quality of classification. To this day the true amount of fatty tissue in the rotator cuff muscles is subject to an estimation by the surgeon, therefore explaining the wide range of interpretation.

To objectively quantify the fat content MR spectroscopic fat measurement was introduced as an experimental method. This technique allows for quantification of fat tissue in a manually applied region of interest by its specific spectroscopic signal. Pfirrmann et al. performed MR spectroscopic fat quantification in a 10 × 10 × 10 mm voxel in the center of the supraspinatus muscle [19]. However, this single-voxel-technique uses cubic voxels and does not cover the whole supraspinatus and thus, may not give the correct water-fat ratio. Kostler et al. introduced the SPLASH (spectroscopic fast low angle shot) technique for exact measurement of the fat/water ratio in the supraspinatus muscle [20]. The SPLASH technique allows quantification of fatty infiltration in an arbitrarily shaped region of interest (ROI) and thus matching the examined region to the individual anatomy which is a great advantage compared to Pfirrmann’s technique. [21] Since SPLASH uses data from standard MR imaging sequences as a basis, intramyocellular lipids should also be assessed since they are part of the acquired signal. However, different compartments of lipids cannot be distinguished but only the total amount of fat inside the region of interest. This technique consists of a series of standard gradient-echo sequences which are available as product-sequences on every scanner from every vendor. Interestingly the feasibility of these objective methods has been shown in various publications in the past, however, they have not been objectively correlated with a clinically used semi quantitative scoring system through different raters implying the interrater and intrarater reliability regarding the comparison of the semi quantitative method with a quantitative method like the MR spectroscopy [19–21].

The purpose of this study was to determine the exact fat ratio in the supraspinatus muscle using spectroscopic measurement and to compare t to the grade of FD evaluated by different raters with the MRI-based Goutallier Classification. We hypothesize that objective methods enable an improvement of the existing classifications and may facilitate the prediction of the clinical outcome.

## Methods

Institutional review board approval was granted and informed consent was obtained from each patient. A statistical power analysis was performed using G*Power 3.1 [22]. The minimal number of patients was set to be *n* = 32 and the minimum number of raters to be *n* = 4 (β > 0.8).

**Fig. 1:**
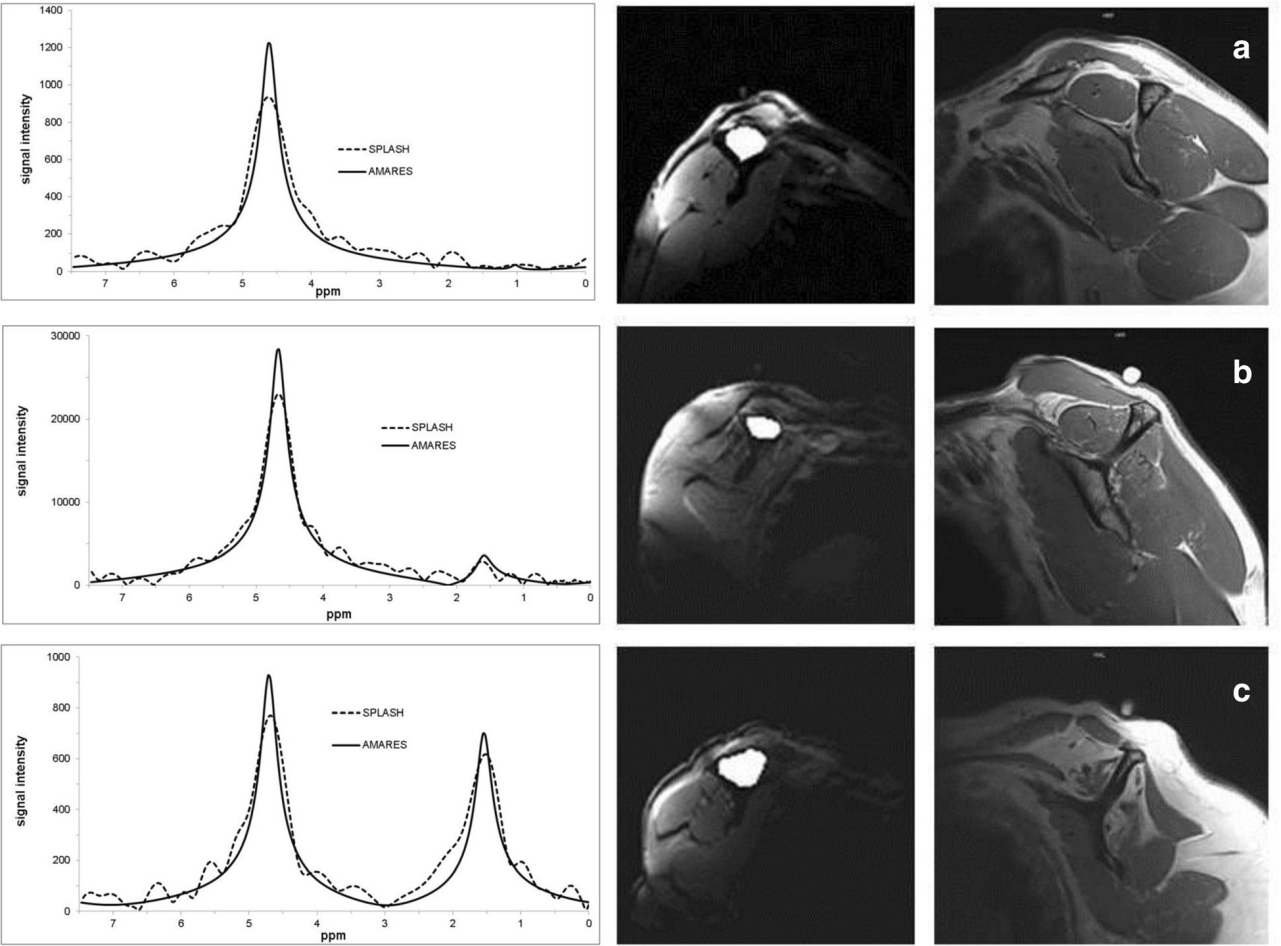
Spectroscopic analysis and quantification of the fat/water ratio in the supraspinatus muscle. The middle column shows the manual delineated borders of the supraspinous fossa in which the spectroscopic analyses were performed. The right collum shwos the accordingly oblique-sagittal t1-weighted MRI images. Calculated values for these examples of the fat /water ratio were **a** 1.29 %, **b** 12.67 % and **c** 77.41 %

Forty-two patients with a history of rotator cuff tear underwent a standard MRI scan of the shoulder. The retraction grade of the supraspinatus tendon was evaluated using the classification according to Patte et al. As part of this MRI scan spectroscopic measurement of the fat/water ratio was performed as described by Kostler et al. [20]. All measurements were performed with a 3 Tesla MRI (Skyra, Siemens, Germany). MRI parameters for the t1-weighted images were: TR = 653 ms, TE = 12 ms, FOV 180 mm and for the SPLASH Sequence: TR = 35 ms, TE = 5–25 ms, FOV 278 mm (TR = repetition time, TE = echo time, FOV = field of view). Slices were 5 mm for the SPLASH technique and 3 mm for the standard MRI. In the evaluation, the muscular borders of the supraspinatus were delineated manually (Fig. 1) and the quantitative evaluation of the spectra was obtained using a home-built reconstruction program (MATLAB 2014b, The MathWorks, Inc., Natick, Massachusetts, United States) as well the time domain fit program AMARES implemented in jMRUI [23].

The manual delineation of the muscle borders was performed by two independent observers.

Semi quantitative assessment of the scans was performed by 5 independent raters according to the MRI-based Goutallier Classification using oblique-sagittal t1-weighted images. Grade 0 was defined as no fatty infiltration, grade 1 as some fatty streaking of the supraspinatus, grade 2 as less fat than muscle, grade 3 as equal amounts of fat and muscle, and grade 4 as more fat than muscle (Table 1). All raters were shoulder fellowship trained orthopedic surgeons (DB, LE, FG, JS, DZ). Three weeks after the first survey all shoulders were rated again in a blinded fashion for evaluation of the intraobserver reliability.

**Table 1:** Semi quantitative scales for estimating fatty degeneration of rotator cuff muscles

	Goutallier et al.	Fuchs et al.	Williams et al.	Slabaugh et al.
no fat	0	1	1	1
fatty streaking	1	2		2
fat < muscle	2		2	
fat = muscle	3	3	3	
fat > muscle	4			3

Comparison of semi quantitative scales for estimating fatty degeneration of rotator cuff muscles [12–14, 17]

The calculated fat/water ratio was correlated with the Goutallier grade of each observer. Correlation was calculated using the Spearman’s rank correlation test. Interobserver and intraobserver reliability was calculated with the same test. Degrees of reliability were set to the scale determined by Landis and Koch [24]. Statistical analysis was performed using SPSS version 14 (IBM, Armonk NY, USA). Results were considered as statistically significant, when *p* < 0.05.

## Results

Forty-two patients with a history of rotator cuff tear and mean age of 58.8 ± 7.65 years (range from 40 to 76) were included in to the study. Clinical data of the patients are shown in Table 2.

**Table 2:** Patients features

Patients’ Features	
Total No. of patients	42
Sex, female:male	29:13
Age [years]	59.8 (±7.7)
Underwent rotator cuff repair	28
Time after surgery [years]	2.3 (±0.7)
Tendon retraction: Patte Classification I:II:III	18:17:7

**Fig. 2:**
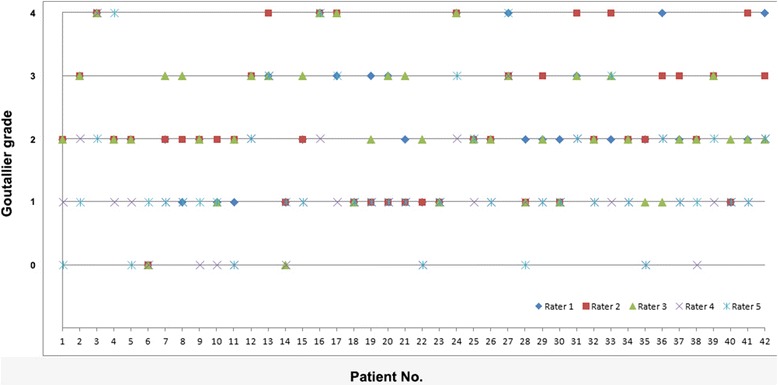
Broad discrepancy of the Goutallier Classification applied by 5 raters for 42 patients. T1-weighted oblique-sagittal images were rated independently according to the modified Goutallier classification established by Fuchs et al. [12]

Mean degree of FD of all supraspinatus muscles, obtained with the SPLASH technique, was 17.9 % ± 18.9 % (mean ± standard deviation) and ranged from 0 to 77 %. The delineation of the muscle borders were performed by two independent observers, showing no differences in the measured spectras, resulting the same amount of FD for each muscle. Therefore it seems conclusive that the measurements of the mr-spectroscpoy are rater independent. Twenty-eight of the patients underwent surgical rotator cuff repair, mean time after surgery was 2.3 years (±0.7). Degrees of reliability were scaled according to the staging determined by Landis and Koch [24]. Statistical analysis of the data revealed a fair correlation of the spectroscopic measured fat ratio and the Goutallier classification system with ρ = 0.35 (*p* < 0.05), the broad variation of the Goutallier classification is illustrated in Fig. 2. By dichotomizing the Goutallier scale (combining group 0–2 and group 3 and 4) the correlation with the spectroscopic fat measurement was ρ = 0.72 (*p* < 0.05). The interobserver reliability was substantial with ρ = 0.62 (*p* < 0.01). The intraobserver agreement was also substantial with ρ = 0.744 and ranged from 0.64 to 0.96 (Table 3). Results of the statistical correlation are shown in Tables 3 and 4.

**Table 3:** Interobserver agreement

	Rater 1	Rater 2	Rater 3	Rater 4	Rater 5	Spectroscopy (SPLASH)
Rater 1	●	0.665**	0.605**	0.779**	0.625**	0.383*
Rater 2	0.665**	●	0.619**	0.609**	0.63**	0.348*
Rater 3	0.605**	0.619**	●	0.609**	0.532**	0.482*
Rater 4	0.779**	0.609**	0.609**	●	0.64**	0.35*
Rater 5	0.625**	0.63**	0.532**	0.64**	●	0.253*
median						0.35

Interobserver agreement and correlation of the SPLASH-technique with the modified Goutallier Classification. The mean R for interobserver reliability was ρ =0.62. Correlation between the modified Goutallier Classification and the spectroscopic fat measurement with the SPLASH technique was ρ = 0.35. Correlation was measured using Spearman’s rank correlation test. (*p < 0.05) (**p < 0.01)

**Table 4:** Intraobserver agreement

	Observer 1	Observer 2	Observer 3	Observer 4	Observer 5
ρ	0.680*	0.646*	0.708*	0.719*	0.967*

Median intraobserver reliability was substantial with *R* = 0.74. The t1-weighted semi-oblique slices were rated a second time after 3 weeks in a randomized order. Correlation was calculated using Spearman's rank correlation test. (*p < 0.05)

## Discussion

The findings of our study demonstrate that there is only a fair correlation (0.34) between the 5-tier Goutallier Classification system in the MRI and the spectroscopic fat measurement using the SPLASH technique, which allows exact quantification of the fat amount in an arbitrarily shaped ROI that covers the whole cross sectional area. Only by dichotomization of the Goutallier scale the correlation can be improved. The interobserver reliability of the MRI-based Goutallier Classification has been found adequate with a k value of 0.62 in a cohort of 5 shoulder trained orthopedic surgeons.

Beside the studies of Kostler et al. [20] and Kenn et al. [21] there is only a single study which investigated MR spectroscopic fat measurement in the rotator cuff. genauer.

Pfirrmann et al. investigated shoulders of 120 patients [19]. These shoulders were graded according to the MRI-based Goutallier Classification and spectroscopic fat measurement was performed. They found that the average fat amount in patients was 19.6 % for Goutallier-grade 0, 36.8 % for -grade 1, 53.6 % for -grade 2, 67.5 % for -grade 3 and 79.2 % for -grade 4. The authors found high amount of fat even in healthy appearing muscles which is a significant contradiction to our findings detecting no or only low fat signals in healthy appearing muscles. Using a voxel for the classification process instead of measuring the whole cross sectional anatomical area may overestimate the fatty infiltration as there are regional incongruities of fatty infiltration in the muscle. A limitation of above mentioned study was the use of a 10 × 10 × 10 mm voxel positioned in the center of the supraspinatus for the spectroscopic analysis. In our study a spatially matched region of interest including the whole supraspinous fossa was used in order to display the (cross sectional) true amount of FD in the complete muscle.

Furthermore, in Pfirrmann’s study the Goutallier Classification was applied by two raters but there is no report about the interobserver reliability between these raters. The level of significance was not reached for distinction between grade 2 and 3 and grade 3 and 4. Nevertheless the study indicates that the Goutallier Classification has weaknesses in the distinction when higher amounts of fat can be found in the muscle tissue. Consequently it only permits to distinguish safely between low and high grades of FD but fails to effectively display the subtle differences in the amount of FD.

Fuchs et al. reported that the MRI classification with the Goutallier system was superior to the CT-based classification with an interobserver agreement of 0.86 for the supraspinatus muscle which may suggest an easy and unanimous applicability of the classification [12]. This study is limited by including only two raters (radiologists). In our cohort of 5 raters presented a distinctively lower interobserver reliability of 0.62 using a comparable imaging technique. This demonstrates the need for a more objective method for FD measurement in order to increase reliability and reproducibility [12].

Multiple studies examined the reliability of the MRI based classification and showed interrater agreements between 0.43 and 0.62 for the 5-tier Goutallier scale. Simplification of the classification was proposed to increase the reliability, but even with simplification the interrater variability remained significant [15–17].

Nevertheless in the clinical setting semi quantitative MRI analysis is the gold standard to determine the amount of FD. However this type of classification yields a high amount of subjective judgment. More quantitative data in the classification process will result in a more accurate classification. Only the incorporation of such aspects in the grading system will lead to a reproducible classification, increase interobserver reliability and thus heighten the level of clinical acceptance for this grading system. It remains speculative if quantitative tools and aspects will then change the approach towards treatment of rotator cuff in general as the current grading system still incorporates substantial amount of estimation. MR spectroscopy may be a useful tool in scientific approaches to evaluate the amount of FD more exactly. Although it has been described in the literature several years ago it has not been implemented into the clinical routine. This may be due to its experimental character on the one hand, as this technique it is only available in a few centers, yet. Once implemented the SPLASH technique can be added to regular MRI imaging of the shoulder with an additional examination time of 3 min. However, one needs the possibility to export the raw data from the MR-scanner in addition to the DICOM-images for further postprocessing. Most vendors are willing to offer this export-ability to their customers when asked.

This is the first study comparing the MRI-based Goutallier Classification system to quantitative fat measurement in a rotator cuff muscle using MR spectroscopic techniques. A limitation of this study is that there is no biological reference of the real fat amount as muscle-biopsies for these investigations remain ethically problematical. Recent studies have shown that FD underlies different pathophysiological mechanisms resulting in muscular atrophy or fatty infiltration. In chronic rotator cuff tears both fat distribution patterns occur. With the present study design we did not differentiate between these patterns of fat distribution. Nevertheless the influence of this on the clinical outcome remains unclear [25, 26]. Additionally the drawn ROIs might include perimuscular fat tissue which is found physiologically and could lead to incorrect fat ratios.

## Conclusion

Quantitative assessment of the fat/water ratio with MR spectroscopic techniques may help to increase the accuracy of predicting clinical results in rotator cuff surgery, as the established grading system does not allow a consistent prediction of the real fat amount in rotator cuff muscles. Clinical studies based on the semi quantitative assessment of FD using the established classification systems must be interpreted carefully. Clinically correlated studies have to prove if exact fat measurement can improve the predictability and may lead to better indications for rotator cuff repair.

## Abbreviations

CT: Computer tomography
FD: Fatty degeneration
FOV: Field of view
mm: Millimeter
MRI: Magnetic resonance imaging
ms: Milliseconds
ROI: Region of interest
SPLASH: Spectroscopic fast low angle shot
TE: Echo time
TR: Repetition time
